# Supplementary material for: Microbial carbon use efficiency promotes global soil carbon storage
Source: Nature. 2023 May 24;618(7967):981–5. doi: 10.1038/s41586-023-06042-3 (PMC10307633; doi:10.1038/s41586-023-06042-3)
Supplement: Supplementary file 1 — This file contains Supplementary discussion, tables, figures and references. [file 41586_2023_6042_MOESM1_ESM.pdf]

nature portfolio

https://doi.org/10.1038/s41586-023-06042-3

Supplementary information

Microbial carbon use efficiency promotes global soil carbon storage

In the format provided by the authors and unedited

Supplementary Information

Microbial carbon use efficiency promotes global soil carbon storage

Feng Tao[1, 2, 3], Yuanyuan Huang[4], Bruce A. Hungate[5, 6], Stefano Manzoni[7], Serita D. Frey[8], Michael W. I. Schmidt[9], Markus Reichstein[2], Nuno Carvalhais[2, 10], Philippe Ciais[11], Lifen Jiang[12], Johannes Lehmann[13], Ying-Ping Wang[14], Benjamin Z. Houlton[15], Bernhard Ahrens[2], Umakant Mishra[16, 17], Gustaf Hugelius[7], Toby D. Hocking[6], Xingjie Lu[18], Zheng Shi[19], Kostiantyn Viatkin[12, 3], Ronald Vargas[3], Yusuf Yigini[3], Christian Omuto[3], Ashish A. Malik[20], Guillermo Peralta[3], Rosa Cuevas-Corona[3], Luciano E. Di Paolo[3], Isabel Luotto[3], Cuijuan Liao[1], Yi-Shuang Liang[1], Vinisa S. Saynes[3], Xiaomeng Huang[1, *], and Yiqi Luo[12, *]

[1]Department of Earth System Science, Ministry of Education Key Laboratory for Earth System Modelling, Institute for Global Change Studies, Tsinghua University, Beijing, China
[2]Max Planck Institute for Biogeochemistry, Jena, Germany
[3]Food and Agricultural Organization of the United Nations, Rome, Italy
[4]Key Laboratory of Ecosystem Network Observation and Modeling, Institute of Geographic Sciences and Natural Resources Research, Chinese Academy of Sciences, Beijing, China.
[5]Center for Ecosystem Science and Society, Department of Biological Sciences, Northern Arizona University, Flagstaff, AZ, USA.
[6]School of Informatics, Computing and Cyber Systems, Northern Arizona University, Flagstaff, AZ, USA
[7]Department of Physical Geography and Bolin Centre for Climate Research, Stockholm University, Stockholm, Sweden
[8]Center for Soil Biogeochemistry and Microbial Ecology, Department of Natural Resources and the Environment, University of New Hampshire, Durham, NH, USA
[9]Department of Geography, University of Zurich, Zurich, Switzerland
[10]Departamento de Ciências e Engenharia do Ambiente, DCEA, Faculdade de Ciências e Tecnologia, FCT, Universidade Nova de Lisboa, Caparica, Portugal
[11]Laboratoire des Sciences du Climat et de l’Environnement, LSCE/IPSL, CEA-CNRS-UVSQ, Université Paris-Saclay, Gif-sur-Yvette, France
[12]School of Integrative Plant Science, Cornell University, Ithaca NY, USA
[13]Soil and Crop Sciences Section, School of Integrative Plant Science, Cornell University, Ithaca NY, USA
[14]CSIRO Environment, Aspendale, Victoria, Australia

[15]Department of Ecology and Evolutionary Biology and Department of Global Development, Cornell University, Ithaca, NY, USA
[16]Computational Biology & Biophysics, Sandia National Laboratories, Livermore, CA, USA
[17]Joint BioEnergy Institute, Lawrence Berkeley National Laboratory, Emeryville, CA, USA
[18]School of Atmospheric Sciences, Sun Yat-sen University, Guangzhou, China
[19]Institute for Environmental Genomics and Department of Microbiology and Plant Biology, University of Oklahoma, Norman, OK, USA
[20]School of Biological Sciences, University of Aberdeen, Aberdeen, UK

*Corresponding Authors: Yiqi Luo: yiqi.luo@cornell.edu; Xiaomeng Huang: hxm@tsinghua.edu.cn

Supplementary Discussion

Analytical solution of SOC storage with the microbial model

Organic carbon dynamics represented by the microbial model can be expressed in a matrix equation[1]:

$$\frac{d\boldsymbol{X}(t)}{dt} = \boldsymbol{B}I(t) + \boldsymbol{A\xi}(t)\boldsymbol{KX}(t) + \boldsymbol{V}(t)\boldsymbol{X}(t) \qquad (S1)$$

We have discussed in the main text that Equation (S1) can be separated into two equations: one for litter carbon cycle and the other for mineral SOC cycle, because there is no carbon transfer from mineral soil carbon pools to litter carbon pools (i.e., $\boldsymbol{a}_{litter\,pool,soil\,pool} = \boldsymbol{0}$ in the ***A*** matrix). Since ***A***, ***K***, $\boldsymbol{\xi}(t)$, and ***V*** are all independent from litter carbon pool states (i.e., ***X***), the analytical solution of litter carbon stock at the steady state (SS) can be calculated as:

$$\boldsymbol{X}_{litter,SS} = \left[\boldsymbol{A}_{litter}\overline{\boldsymbol{\xi}(t)_{litter}}\boldsymbol{K}_{litter} + \overline{\boldsymbol{V}(t)_{litter}}\right]^{-\boldsymbol{1}}\left[-\boldsymbol{B}_{litter}\overline{\boldsymbol{I}(t)_{litter}}\right] \qquad (S2)$$

For the mineral soil part, the related ***K*** matrix is carbon pool state dependent (Equation 5 of the main text). The steady-state solution for the mineral soil organic carbon pools cannot be readily obtained by an equation similar with Equation (S2). Thus, we transformed the matrix equation for the mineral soil carbon pools into four differential equations for each of the 20 soil layers:

$$\frac{dx_{DOC}}{dt} = u_{DOC} + \frac{v_{max,decom}\xi x_{ENZ}x_{mSOC}}{K_{m,decom}\xi + x_{mSOC}} + a_{DOC,MIC}k_{MIC}\xi x_{MIC} + k_{ENZ}\xi x_{ENZ} - \frac{v_{max,assim}\xi x_{MIC}x_{DOC}}{K_{m,assim}\xi + x_{DOC}} \qquad (S3)$$

$$\frac{dx_{MIC}}{dt} = u_{MIC} + \eta_{DOC}\frac{v_{max,assim}\xi x_{MIC}x_{DOC}}{K_{m,assim}\xi + x_{DOC}} - k_{MIC}\xi x_{MIC} \qquad (S4)$$

$$\frac{dx_{ENZ}}{dt} = a_{ENZ,MIC}k_{MIC}\xi x_{MIC} - k_{ENZ}\xi x_{ENZ} \qquad (S5)$$

$$\frac{dx_{mSOC}}{dt} = u_{mSOC} + a_{mSOC,MIC}k_{MIC}\xi x_{MIC} - \frac{v_{max,decom}\xi x_{ENZ}x_{mSOC}}{K_{m,decom}\xi + x_{mSOC}} \qquad (S6)$$

Where DOC, MIC, ENZ, mSOC are the four soil carbon pools for dissolved organic carbon, microbial organic carbon, enzyme organic carbon, and mineral-associated soil organic carbon, respectively. u_{S_i} is the carbon input from litter pools (L_j) to a mineral soil carbon pool (S_i, see Extended Data Fig. 3 for corresponding carbon flows for each mineral soil carbon pool) and can be expressed as $\sum_{L_j}\left(a_{S_i,L_j}k_{L_j}\xi x_{L_j}\right)$. η_{DOC} is the microbial carbon use efficiency for DOC and equals $a_{MIC,DOC}$ in the $\boldsymbol{A}$ matrix (see Equation 6 in the main text).

At steady state, equations (S3) to (S6) equal 0 (i.e., $\frac{dx}{dt}=0$). From (S4), we have:

$$k_{MIC}\xi x_{MIC} = u_{MIC} + \eta_{DOC}\frac{v_{max,assim}\xi x_{MIC}x_{DOC}}{K_{m,assim}\xi + x_{DOC}} \qquad (S7)$$

(S3) + (S6) gives:

$$u_{mSOC} + u_{DOC} + \left(a_{DOC,MIC} + a_{mSOC,MIC}\right)k_{MIC}\xi x_{MIC} + k_{ENZ}\xi x_{ENZ} = \frac{v_{max,assim}\xi x_{MIC}x_{DOC}}{K_{m,assim}\xi + x_{DOC}} \qquad (S8)$$

Equation (S5) at the steady state gives:

$$a_{ENZ,MIC}k_{MIC}\xi x_{MIC} = k_{ENZ}\xi x_{ENZ} \qquad (S9)$$

Substitute (S9) into (S8) and then substitute (S8) into the right part of (S7):

$$k_{MIC}\xi x_{MIC} = u_{MIC} + \eta_{DOC}\left[u_{mSOC} + u_{DOC} + \left(a_{DOC,MIC} + a_{mSOC,MIC}\right)k_{MIC}\xi x_{MIC} + k_{ENZ}\xi x_{ENZ}\right] = u_{MIC} + \eta_{DOC}(u_{mSOC} + u_{DOC} + k_{MIC}\xi x_{MIC}) \qquad (S10)$$

Thus, we have MIC at steady state:

$$x_{MIC,SS} = \frac{u_{MIC} + \eta_{DOC}(u_{mSOC} + u_{DOC})}{(1-\eta_{DOC})k_{MIC}\xi} \qquad (S11)$$

From (S9), we have ENZ at the steady state:

$$x_{ENZ,SS} = \frac{a_{ENZ,MIC} k_{MIC} x_{MIC,SS}}{k_{ENZ}} \qquad (S12)$$

At the steady state, Equation (S6) gives the steady state solution for mSOC:

$$x_{mSOC,SS} = \frac{\left(u_{mSOC} + a_{mSOC,MIC} k_{MIC} \xi x_{MIC,SS}\right) K_{m,decom} \xi}{\left(v_{max,decom} \xi x_{ENZ,SS} - a_{mSOC,MIC} k_{MIC} \xi x_{MIC,SS} - u_{mSOC}\right)} \qquad (S13)$$

Similarly, Equation (S3) at steady state gives the steady state solution for DOC:

$$x_{DOC,SS} = \frac{k_{MIC} \xi K_{m,assim} \xi x_{MIC,SS} - u_{MIC} K_{m,assim} \xi}{(\eta_{DOC} v_{max,assim} - k_{MIC}) \xi x_{MIC,SS} + u_{MIC}} \qquad (S14)$$

Putting Equations (S11 - S14) together, the analytical solution for the mineral soil organic carbon pools is:

$$\boldsymbol{X}_{soil,SS} = \begin{bmatrix} \boldsymbol{x}_{DOC,SS} \\ \boldsymbol{x}_{MIC,SS} \\ \boldsymbol{x}_{ENZ,SS} \\ \boldsymbol{x}_{mSOC,SS} \end{bmatrix} = \begin{bmatrix} \dfrac{\boldsymbol{k}_{MIC} \boldsymbol{\xi} K_{m,assim} \boldsymbol{\xi} \boldsymbol{x}_{MIC,SS} - \boldsymbol{u}_{MIC} K_{m,assim} \boldsymbol{\xi}}{(\eta_{DOC} v_{max,assim} - \boldsymbol{k}_{MIC}) \boldsymbol{\xi} \boldsymbol{x}_{MIC,SS} + \boldsymbol{u}_{MIC}} \\ \dfrac{\boldsymbol{u}_{MIC} + \eta_{DOC}(\boldsymbol{u}_{mSOC} + \boldsymbol{u}_{DOC})}{(1 - \eta_{DOC}) \boldsymbol{k}_{MIC} \boldsymbol{\xi}} \\ \dfrac{\boldsymbol{a}_{ENZ,MIC} \boldsymbol{k}_{MIC} \boldsymbol{x}_{MIC,SS}}{\boldsymbol{k}_{ENZ}} \\ \dfrac{\left(\boldsymbol{u}_{mSOC} + \boldsymbol{a}_{mSOC,MIC} \boldsymbol{k}_{MIC} \boldsymbol{\xi} \boldsymbol{x}_{MIC,SS}\right) K_{m,decom} \boldsymbol{\xi}}{\left(v_{max,decom} \boldsymbol{\xi} \boldsymbol{x}_{ENZ,SS} - \boldsymbol{a}_{mSOC,MIC} \boldsymbol{k}_{MIC} \boldsymbol{\xi} \boldsymbol{x}_{MIC,SS} - \boldsymbol{u}_{mSOC}\right)} \end{bmatrix} \qquad (S15)$$

where all the elements with bold font indicate vectors of the corresponding variables or parameters for the 20 soil layers. All the multiplications shown in Equation (S15) are element-wise operations.

Supplementary Tables and Figures

Supplementary Table 1 | Summary of CUE and SOC data used in the meta-analysis.

Site ID	Longitude	Latitude	MAT (℃)	Depth (cm)	CUE	Microbial biomass (mg/kg)	SOC (g/kg)	Method	Source
1	118.7083	33.9000	13.4	10	0.1400	256	15.82	^{18}O	ref[2]
2	125.3000	51.2000	-2.4	5	0.5500	2070	69.7		ref[3]
3	121.8000	48.7000	-1	5	0.3700	1490	49.2		
4	128.8000	47.2000	0	5	0.4200	2380	108.7		
5	127.5000	45.3000	2.8	5	0.1000	990	40.7		
6	127.6000	42.7000	3.6	5	0.6300	3780	131.5		
7	124.9000	41.9000	4.7	5	0.2600	1210	53.1		
8	115.4000	40.0000	5	5	0.4300	760	45.2		
9	117.1000	36.3000	4.9	5	0.3400	440	16.9		
10	107.1000	31.9000	13.1	5	0.1700	400	15.6		
11	114.0000	32.1000	15.2	5	0.0800	170	32.3		
12	114.0000	32.1000	16	5	0.2900	580	16.7		
13	118.0000	26.8000	20	5	0.1000	480	29.7		
14	109.6000	26.9000	16.5	5	0.1600	600	29.4		
15	112.5000	23.1000	20.9	5	0.1600	830	26.8		
16	108.9000	18.7000	19.8	5	0.2000	430	24.7		
17	108.8000	18.7000	19.8	5	0.0800	130	18.3		
18	113.8500	35.1500	13.9	7.5	0.0400		9.37	^{13}C	ref[4]
18	113.8500	35.1500	13.9	7.5	0.0400		11.12		
18	113.8500	35.1500	13.9	7.5	0.0505		13.52		
19	120.5000	31.5000	16	7.5	0.0467		27.94		
19	120.5000	31.5000	16	7.5	0.0427		30.47		
19	120.5000	31.5000	16	7.5	0.0517		33.63		
20	11.6222	49.9706	8.4	1.5	0.2290	748	33.7	^{18}O	ref[5]
20	11.6222	49.9706	8.4	5	0.4000	478	24.6		
20	11.6222	49.9706	8.4	12.5	0.2190	204	16.3		
20	11.6222	49.9706	8.4	37.5	0.0782	40	5.9		
21	11.5825	49.9756	8.1	1.5	0.2190	109	26.6		
21	11.5825	49.9756	8.1	5	0.2490	45	6.9		
21	11.5825	49.9756	8.1	12.5	0.3290	33	3.3		
21	11.5825	49.9756	8.1	37.5	0.3090	17	1.5		
22	11.5886	49.9722	8.2	1.5	0.1890	69	11.7		
22	11.5886	49.9722	8.2	5	0.2090	35	6.6		
22	11.5886	49.9722	8.2	12.5	0.2090	28	4.8		
22	11.5886	49.9722	8.2	37.5	0.1990	29	3.9		
23	14.1028	49.4936	7	3.5	0.3090	1177	38.4		ref[6]
23	14.1028	49.4936	7	3.5	0.3200	1052	33.7		
23	14.1028	49.4936	7	3.5	0.4510	1368	43.9		
23	14.1028	49.4936	7	3.5	0.4110	902	33.2		
23	14.1028	49.4936	7	3.5	0.4300	1064	34.4		
24	11.0728	51.5669	6.5	7.5	0.2845		42		ref[7]
25	11.9019	52.7808	9	7.5	0.3055		13		
26	116.2833	42.0333	2.1	7.5	0.5004		16.94	^{13}C	ref[8]
27	9.9094	51.3808	10.5	7.5	0.3300		11.4		ref[9]
28	77.5664	13.0900	24	7.5	0.4200		8.9		
29	14.1000	47.4833	7.2	7.5	0.3598	292	21.8		ref[10]
29	14.1000	47.4833	7.2	7.5	0.4995	647	26.7		
29	14.1000	47.4833	7.2	7.5	0.6480	581	49.9		
30	14.0667	47.5000	7.2	7.5	0.4091	1441	47		

30	14.0667	47.5000	7.2	7.5	0.3895	1809	47.9		
30	14.0667	47.5000	7.2	7.5	0.2498	890	36.8		
31	-21.1858	64.0003	11	5	0.2455	1228	60.98		
31	-21.1858	64.0003	11.5	5	0.2579	1061	59.16		
31	-21.1858	64.0003	12.5	5	0.2006	627	36.5		ref[11]
31	-21.1858	64.0003	14	5	0.2436	838	45.17		
31	-21.1858	64.0003	17	5	0.2292	850	37.34		
32	112.3000	28.1167	17.2	7.5	0.4775	945	19.73		
32	112.3000	28.1167	17.2	7.5	0.4925	1137	20.34		
32	112.3000	28.1167	17.2	7.5	0.4795	1302	24.39		ref[12]
32	112.3000	28.1167	17.2	7.5	0.4855	1313	28.77		
32	112.3000	28.1167	17.2	7.5	0.4650	1808	33.09		
33	-93.2100	45.4300	6	7.5	0.2102		9.4		
33	-93.2100	45.4300	6	7.5	0.2397		15.7		
33	-93.2100	45.4300	6	7.5	0.2923		9		
33	-93.2100	45.4300	6	7.5	0.2593		11		
33	-93.2100	45.4300	6	22.5	0.2973		5.2		
33	-93.2100	45.4300	6	22.5	0.3612		10.4		
33	-93.2100	45.4300	6	22.5	0.3160		4.4		
33	-93.2100	45.4300	6	22.5	0.1752		5.8		
34	-93.3900	41.7900	9	7.5	0.3330		7.2		
34	-93.3900	41.7900	9	7.5	0.3948		8.2		
34	-93.3900	41.7900	9	7.5	0.4079		6.9		
34	-93.3900	41.7900	9	7.5	0.4172		7.4		
34	-93.3900	41.7900	9	22.5	0.4649		4.1		
34	-93.3900	41.7900	9	22.5	0.3503		5.1		
34	-93.3900	41.7900	9	22.5	0.3501		4		
34	-93.3900	41.7900	9	22.5	0.4217		4.1		
35	-0.6400	51.4100	10	7.5	0.5630		24.3	^{18}O	
35	-0.6400	51.4100	10	7.5	0.5174		28.7		
35	-0.6400	51.4100	10	7.5	0.4939		26.9		
35	-0.6400	51.4100	10	7.5	0.4516		24.9		
35	-0.6400	51.4100	10	22.5	0.5056		10.5		
35	-0.6400	51.4100	10	22.5	0.4624		12.8		ref[13]
35	-0.6400	51.4100	10	22.5	0.4358		11.6		
35	-0.6400	51.4100	10	22.5	0.4305		10.1		
36	-0.6400	51.4100	10	7.5	0.4420		36.7		
36	-0.6400	51.4100	10	7.5	0.4837		36.7		
36	-0.6400	51.4100	10	7.5	0.4742		36.5		
36	-0.6400	51.4100	10	7.5	0.4515		37		
36	-0.6400	51.4100	10	22.5	0.5321		24.4		
36	-0.6400	51.4100	10	22.5	0.5822		24.5		
36	-0.6400	51.4100	10	22.5	0.6202		25.6		
36	-0.6400	51.4100	10	22.5	0.5299		23.9		
37	30.4000	-29.6700	18	7.5	0.2791		42		
37	30.4000	-29.6700	18	7.5	0.2504		42.5		
37	30.4000	-29.6700	18	7.5	0.2779		44.4		
37	30.4000	-29.6700	18	7.5	0.2407		45.7		
37	30.4000	-29.6700	18	22.5	0.3435		37.5		
37	30.4000	-29.6700	18	22.5	0.3635		32		
37	30.4000	-29.6700	18	22.5	0.3974		34.8		
37	30.4000	-29.6700	18	22.5	0.3593		36.4		
38	30.7200	-29.8100	18	7.5	0.4848		49.1		
38	30.7200	-29.8100	18	7.5	0.5516		51.1		
38	30.7200	-29.8100	18	7.5	0.4151		51.7		

38	30.7200	-29.8100	18	7.5	0.4746		51.7		
39	112.1000	28.3667	17	5	0.1316	740	18.36		
39	112.1000	28.3667	17	15	0.0760	352	15.77		
39	112.1000	28.3667	17	25	0.0660	124	6.44		
39	112.1000	28.3667	17	5	0.1127	800	17.46		
39	112.1000	28.3667	17	15	0.1480	528	16.11		ref[14]
39	112.1000	28.3667	17	25	0.1850	222	9.39		
39	112.1000	28.3667	17	5	0.1384	970	25.43		
39	112.1000	28.3667	17	15	0.1540	870	24.98		
39	112.1000	28.3667	17	25	0.2285	446	12.09		
40	-123.8103	39.3753	10.8	10	0.4900		100		
40	-123.8103	39.3753	10.8	6.5	0.4600		140		
40	-123.8103	39.3753	10.8	1.5	0.6100		67.5	^{14}C	ref[15]
40	-123.8103	39.3753	10.8	1.5	0.5100		54		
40	-123.8103	39.3753	10.8	2	0.6700		43		
41	127.6300	42.7000	4	5	0.1702	1876	33.91		
41	127.6300	42.7000	4	5	0.2101	1236	39.44		ref[16]
41	127.6300	42.7000	4	5	0.3900	1422	49.73		
42	10.4195	52.2840	9.3	5	0.5900		16.6		
42	10.4195	52.2840	9.3	5	0.2400		12.3		
43	9.9962	52.2009	9.3	5	0.2400		43.3		
43	9.9962	52.2009	9.3	5	0.2800		22.7	^{18}O	
44	10.6065	52.3304	9.2	5	0.1800		24.2		
44	10.6065	52.3304	9.2	5	0.1500		19		ref[17]
45	10.5238	52.3882	9.1	5	0.7900		64.1		
45	10.5238	52.3882	9.1	5	0.0900		18.6		
46	10.4354	52.2998	9.2	5	0.4500		13.7		
46	10.4354	52.2998	9.2	5	0.4200		7.7		

Supplementary Table 2 | Unstandardized coefficients of relationships between CUE and microbial and non-microbial biomass in the mixed-effects model with meta-analysis data. CUE, depth and mean annual temperature (MAT) were set as the fixed effects to microbial and non-microbial biomass carbon content (i.e., total SOC minus microbial biomass carbon). The study source was set as the random effect. We assumed random intercepts in all regressions. The total observation size n_{sample} = 62; the random effects size n_{study} = 9.

		Intercept	CUE	Depth	MAT
$Microbial\ biomass \sim CUE + Depth + MAT + (1 \mid Study\ Source)$<br>variance explained by mixed model: 63%					
Fixed Effects	Estimates	0.79	2.01	-0.011	-0.038
	Std. Error	0.32	0.58	0.0080	0.014
	t value	2.48	3.47	-1.37	-2.66
	P	0.018	0.0011	0.18	0.010
Random Effects	Standard Deviation	0.34	NA	NA	NA
$Nonmicrobial\ biomass \sim CUE + Depth + MAT + (1 \mid Study\ Source)$<br>variance explained by mixed model: 67%					
Fixed Effects	Estimates	31.68	60.56	-0.45	-1.42
	Std. Error	10.16	18.21	0.25	0.45
	t value	3.12	3.33	-1.80	-3.14
	P	0.0034	0.0016	0.077	0.0027
Random Effects	Standard Deviation	11.39	NA	NA	NA

Supplementary Table 3 | Unstandardized coefficients of the CUE-SOC relationship (considering fixed effects from other covariates) in the mixed-effects model with microbial model data assimilation results. CUE and one other environmental variable (i.e., bulk density, citation exchange capacity, clay content, or NPP) were set as the fixed effects to logarithmic SOC content. Climate types that soil profiles belong to were set as the random effect. We applied a mixed-effects model that considered random intercepts with random slopes to test CUE-SOC relationship (i.e., $\mathrm{log10(SOC){\sim}CUE + Selected\ Variable + (CUE|Climate\ Types)}$). The random effects size $\mathrm{n_{climate}} = 12$. The total observation size $n_{obs} = 56{,}270$. The observation size is different from the total soil profile size (i.e., 57,267) because the environmental variable or climate type information is not available for some profiles.

		Intercept	CUE	Variable
$\log(SOC){\sim}CUE + \log(Bulk\ Density) + (CUE|Climate\ Types)$, explained variation = 42%				
Fixed Effects	Estimates	5.92	0.95	-1.61
	Std. Error	0.052	0.081	0.011
	t value	114.62	11.61	-147.75
	P	<0.0001	<0.0001	<0.0001
Random Effects	Standard Deviation	0.13	0.28	NA
$\log(SOC){\sim}CUE + CEC + (CUE|Climate\ Types)$, explained variation = 31%				
Fixed Effects	Estimates	0.64	1.00	0.014
	Std. Error	0.048	0.087	0.00011
	t value	13.33	11.52	122.41
	P	<0.0001	<0.0001	<0.0001
Random Effects	Standard Deviation	0.17	0.30	NA
$\log(SOC){\sim}CUE + Clay\ Content + (CUE|Climate\ Types)$, explained variation = 18%				
Fixed Effects	Estimates	0.76	1.11	0.0067
	Std. Error	0.056	0.13	0.00013
	t value	13.50	8.71	52.83
	P	<0.0001	<0.0001	<0.0001
Random Effects	Standard Deviation	0.19	0.44	NA
$\log(SOC){\sim}CUE + \log(NPP) + (CUE|Climate\ Types)$, explained variation = 18%				
Fixed Effects	Estimates	0.84	1.11	0.029
	Std. Error	0.057	0.13	0.0054
	t value	14.64	8.78	5.31
	P	<0.0001	<0.0001	<0.0001
Random Effects	Standard Deviation	0.19	0.43	NA

Supplementary Table 4 | Unstandardized coefficients of relationships between CUE and microbial and non-microbial biomass in the mixed-effects model with microbial model data assimilation results. CUE was set as the fixed effects to microbial and non-microbial biomass (i.e., total SOC minus microbial biomass carbon) carbon content. Climate types that soil profiles belong to were set as the random effect. We assumed random intercepts in all regressions. The total observation size n_{obs} = 56,270, the random effects size $\mathrm{n_{climate}}$ = 12. The observation size is different from the soil profile size used in microbial model data assimilation (i.e., 57,267) because the climate type information is not available for some profiles.

		Intercept	CUE
$Microbial\ biomass \sim CUE + (1\|Climate\ Type)$<br>variance explained by mixed model: 29%			
Fixed Effects	Estimates	-0.95	10.07
	Std. Error	0.38	0.096
	t value	-2.51	104.44
	P	0.029	<0.0001
Random Effects	Standard Deviation	1.31	NA
$Nonmicrobial\ biomass \sim CUE + (1\|Climate\ Type)$<br>variance explained by mixed model: 17%			
Fixed Effects	Estimates	4.23	64.15
	Std. Error	3.51	0.86
	t value	1.21	74.96
	P	0.25	<0.0001
Random Effects	Standard Deviation	12.12	NA

Supplementary Table 5 | Relationship between PRODA-retrieved CUE values in these pixels where CUE was measured in the meta-analysis and the measured values. CUE predicted by the PRODA approach (CUE_{proda}) was set as the fixed effects to measurements in the meta-analysis (CUE_{meta}). The study source was set as the random effect. We assumed random intercepts in the regression. The total observation size n_{sample} = 132; the random effects size n_{study} = 16. The difference of the regression slope between CUE_{meta} and CUE_{proda} from 1 was tested by offsetting CUE_{proda} in the same mix-effects model.

		Intercept	CUE_{proda}
$CUE_{meta} \sim CUE_{proda} + (1\|Study\ Source)$, R^2 = 0.54			
Fixed Effects	Estimates	0.14	0.66
	Std. Error	0.068	0.22
	t value	1.99	3.01
	P	0.050	0.0032
Random Effects	Standard Deviation	0.11	NA
$CUE_{meta} \sim CUE_{proda} + 1 * CUE_{proda} + (1\|Study\ Source)$			
Fixed Effects	Estimates	0.14	-0.34
	Std. Error	0.068	0.22
	t value	1.99	-1.56
	P	0.050	0.12
Random Effects	Standard Deviation	0.11	NA

Supplementary Table 6 | Parameters in the vertically-resolved microbial model that were optimized in the profile-level data assimilation.

No.	Name	Related components	Description	Conventional values	Unit	Prior range
1	η_{DOC}	Microbial carbon use efficiency	Microbial CUE for DOC assimilation	0.6	unitless	[0.01 0.7]
2	η_{ML}		Microbial CUE for metabolic litter assimilation	0.5	unitless	[0.4, 0.9]
3	η_{CL-LL}		Microbial CUE for cellulose/lignin litter assimilation	0.5	unitless	[0, 0.4]
4	$K_{m,assim}$		Concentration of DOC for half max DOC assimilation reaction	4×10^2	gCm^{-3}	[300 3000]
5	τ_{assim}		Inverse of $v_{max,assim}$ in DOC assimilation	0.011	year	[0.03 0.001]
6	τ_{decom}	Decomposition	Inverse of $v_{max,decom}$ in SOC decomposition	1.1×10^{-4}, 4.6×10^{-5}, 2×10^{-7}	year	[0 3×10^{-4}]
7	$K_{m,decom}$		Concentration of SOC for half max SOC decomposition reaction	6×10^5	gCm^{-3}	[10^5 10^6]
8	$\tau_{ENZ,prod}$		Turnover time for enzyme production	22	year	[15 30]
9	τ_{ML}		Turnover time of metabolic litter	0.0541	year	[0 0.1]
10	τ_{CWD}		Turnover time of coarse woody debris	3.33	year	[1 6]
11	τ_{CL-LL}		Turnover time of cellulose and lignin litter	0.2041	year	[0.1 0.3]
12	$\tau_{ENZ,decay}$		Turnover time for enzyme decay	0.11	year	[0.001 1]
13	τ_{MIC}		Turnover time for microbial mortality	0.57	year	[0 2]
14	$a_{SOC,MIC}$	Carbon transfer fraction	Fraction of microbial necromass that is stabilized as SOC	0.5	year	[0 1]
15	$a_{CL,CWD}$		Fraction of decomposed CWD that goes to cellulose litter	0.75	unitless	[0.5, 1]
16	$a_{DOC,ML}$		Fraction of total decomposed metabolic litter that goes to DOC	0.05	unitless	[0 0.1]
17	$a_{DOC,CL}$		Fraction of total decomposed cellulose litter that goes to DOC	0.15	unitless	[0.05 0.3]
18	$a_{SOC,LL}$		Fraction of total decomposed cellulose litter that goes to SOC	0.8	unitless	[0.6 0.95]
19	*w-scaling*	Environmental modification	Scaling factor to soil water scalar	1	unitless	[0 5]
20	*q10*		Temperature sensitivity	1.5	unitless	[1.2 3]
21	*cryo*	Vertical transport	Cryoturbation rate	0.0005	m^2yr^{-1}	[3×10^{-5} 16×10^{-4}]
22	*diffus*		Bioturbation rate	0.0001	m^2yr^{-1}	[3×10^{-5} 5×10^{-4}]
23	*b*	Carbon input allocation	Parameter controlling vertical distribution of carbon input to litter pools	PFT dependent	unitless	[0.5 1]

Supplementary Table 7 | Unstandardized coefficients of the CUE-SOC relationship in the mixed-effects model with different isotope-derived CUE data in the meta-analysis. CUE, depth and mean annual temperature (MAT) were set as the fixed effects to SOC content. The study source was set as the random effect.

		Intercept	CUE	Depth	MAT
$^{13}C/^{14}C$ derived relationship, $n_{obs} = 21$, $n_{study} = 6$<br>$SOC \sim CUE + Depth + MAT + (1\|Study\ Source)$, explained variation = 79%					
Fixed Effects	Estimates	-22.67	**16.28**	6.61	0.04
	Std. Error	36.91	47.14	2.50	1.61
	t value	-0.64	0.35	2.61	0.026
	P	0.55	0.73	0.020	0.98
Random Effects	Standard Deviation	36.33	NA	NA	NA
^{18}O derived relationship, $n_{obs} = 111$, $n_{study} = 10$<br>$SOC \sim CUE + Depth + MAT + (1\|Study\ Source)$, explained variation = 46%					
Fixed Effects	Estimates	14.99	**61.06**	-0.72	0.17
	Std. Error	7.33	12.56	0.21	0.35
	t value	2.04	4.86	-3.46	0.48
	P	0.046	<0.0001	0.0007	0.63
Random Effects	Standard Deviation	10.51	NA	NA	NA

Supplementary Table 8 | Forcing variables for driving the microbial model simulation

Variable Names	Full Description	Resolution
nbedrock	Soil layer number that reaches the bedrock	0.5 degree, monthly record of 20-year simulation after the system reaches the steady state.
ALTMAX	Maximum active layer depth of current year	
ALTMAX_LASTYEAR	Maximum active layer depth of last year	
CELLSAND	Sand content	
NPP	Net primary productivity	
SOILPSI	Soil water potential	
TSOI	Soil temperature	
O_SCALAR	Oxygen scalar for decomposition	
FPI_vr	Nitrogen scalar for decomposition	

These forcing variables were the outputs from CLM5 simulation.

Supplementary Table 9 | Environmental variables used in predicting optimized parameter values of the microbial model by the deep learning model. Note that information of some of the variables (e.g., clay content) was reported at different depths (i.e., 0cm, 30cm, and 100cm).

No.	Variable Name	Data Source	Category	Description
1	Longitude	WoSIS	Geography	
2	Latitude	WoSIS		
3	Elevation	NOAA		Available at https://www.ngdc.noaa.gov/mgg/global/
4	Absolute Depth to Bedrock	ref[18]		
5	Bedrock Depth	CLM5 simulation		
6	Koppen Climate Classification	ref[19]	Climate	
7	Annual Mean Temperature	ref[20]		
8	Mean Diurnal Range Temperature			
9	Isothermality			
10	Temperature Seasonality			
11	Max Temperature of Warmest Month			
12	Min Temperature of Coldest Month			
13	Temperature Annual Range			
14	Mean Temperature of Wettest Quarter			
15	Mean Temperature of Driest Quarter			
16	Mean Temperature of Warmest Quarter			
17	Mean Temperature of Coldest Quarter			
18	Annual Precipitation			
19	Precipitation of Wettest Month			
20	Precipitation of Driest Month			
21	Precipitation Seasonality			
22	Precipitation of Wettest Quarter			
23	Precipitation of Driest Quarter			
24	Precipitation of Warmest Quarter			
25	Precipitation of Coldest Quarter			
26	USDA 2014 Suborder Classes	ref[18]	Soil Structure	
27	WRB 2006 Subgroup Classes			
28	Coarse Fragments Volumetric			Three depths were included, which are 0cm, 30cm and 100cm, respectively
29	Clay Content			Three depths were included, which are 0cm, 30cm and 100cm, respectively
30	Silt Content			Three depths were included, which are 0cm, 30cm and 100cm, respectively
31	Texture Classes			Three depths were included, which are 0cm, 30cm and 100cm, respectively
32	Sand Content			Three depths were included, which are 0cm, 30cm and 100cm, respectively
33	Bulk Density			Three depths were included, which are 0cm, 30cm and 100cm, respectively
34	Soil Water Capacity			Three depths were included, which are 0cm, 30cm and 100cm, respectively
35	Soil pH in H_2O	ref[18]		Three depths were included, which are 0cm, 30cm and 100cm, respectively

36	Soil pH		Soil Chemical Properties	Three depths were included, which are 0cm, 30cm and 100cm, respectively
37	Cation Exchange Capacity			Three depths were included, which are 0cm, 30cm and 100cm, respectively
38	Grade of a Sub-Soil Being Acid			
39	ESA Land Cover	ESA. Land Cover CCI Product User Guide Version 2. Tech. Rep. (2017).	Vegetation	Available at: maps.elie.ucl.ac.be/CCI/viewer/download/ESACCI-LC-Ph2-PUGv2_2.0.pdf
40	NPP	CLM5 simulation		Mean value of 20-year simulation after the system reaches the steady state
41	Standard deviation of NPP			Standard deviation of 20-year simulation after the system reaches the steady state
42	Vegetation Carbon Stock			Mean value of 20-year simulation after the system reaches the steady state

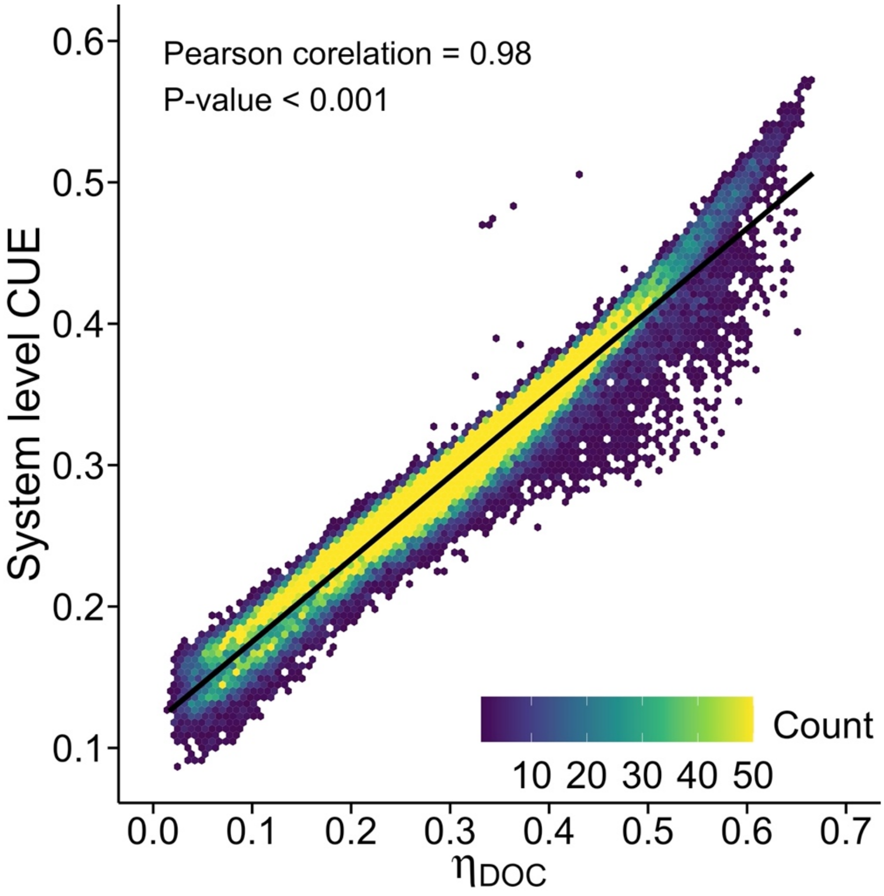

Supplementary Fig. 1 | Dependence of system-level CUE on the CUE of the mineral soil part.

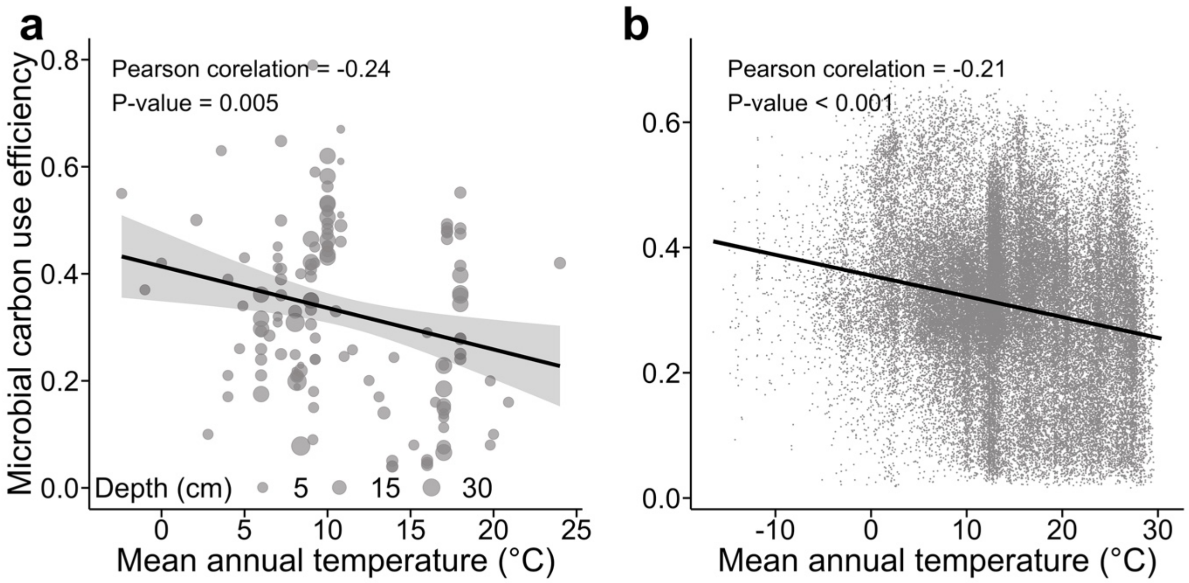

Supplementary Fig. 2 | The MAT-CUE relationship emerged from the meta-analysis with 132 measurements (a) and microbial model data assimilation with all 57,267 profiles (b). Black lines and shaded areas are the linear regression results and 95% confidence interval.

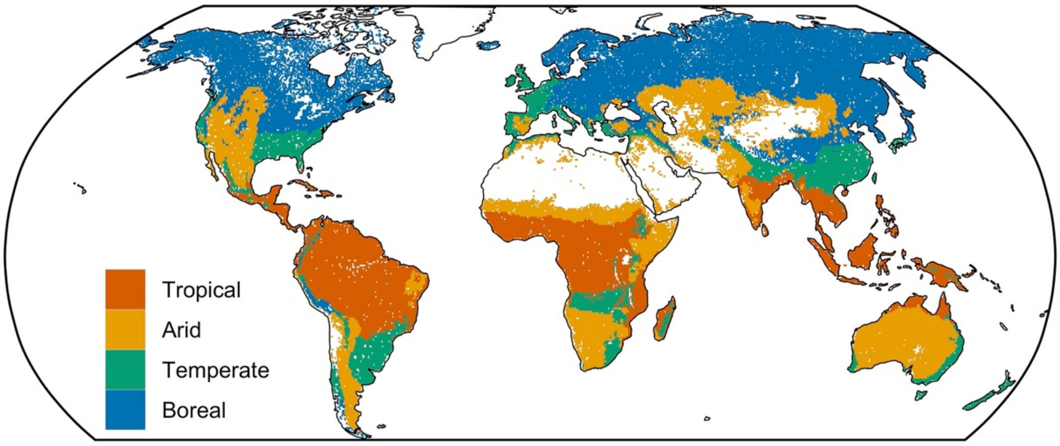

Supplementary Fig. 3 | Distribution of climate zones. Data is taken from ref[19].

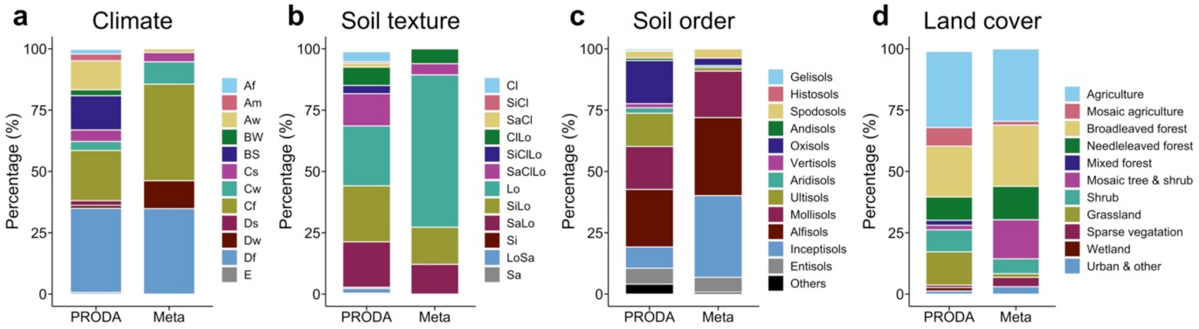

Supplementary Fig. 4 | Coverage of different sources of data in multi-dimensional covariate spaces. Panels show the percentage of data sites located at different climates (a), soil textures (b) soil orders (c), and land cover types (d) in the profiles used in the PRODA approach of this study (i.e., 57,267 profiles) and the meta-analysis with 132 data sets. For different climate types: Af, Am and Aw are tropical rainforest, monsoon and savannah climates, respectively. BW and BS are arid desert and steppe climates, respectively. Cs, Cw and Cf are temperate climates with dry summer, dry winter, and without dry season, respectively. Ds, Dw and Df are cold climates with dry summer, dry winter, and without dry season, respectively. E is polar climate. For different soil texture, Cl is clay, SiCl is silty clay, SaCl is sandy clay, ClLo is clay loam, SiClLo is silty clay loam, SaClLo is sandy clay loam, Lo is loam, SiLo is silty loam, SaLo is sandy loam, Si is silt, LoSa is loamy sand, Sa is sand.

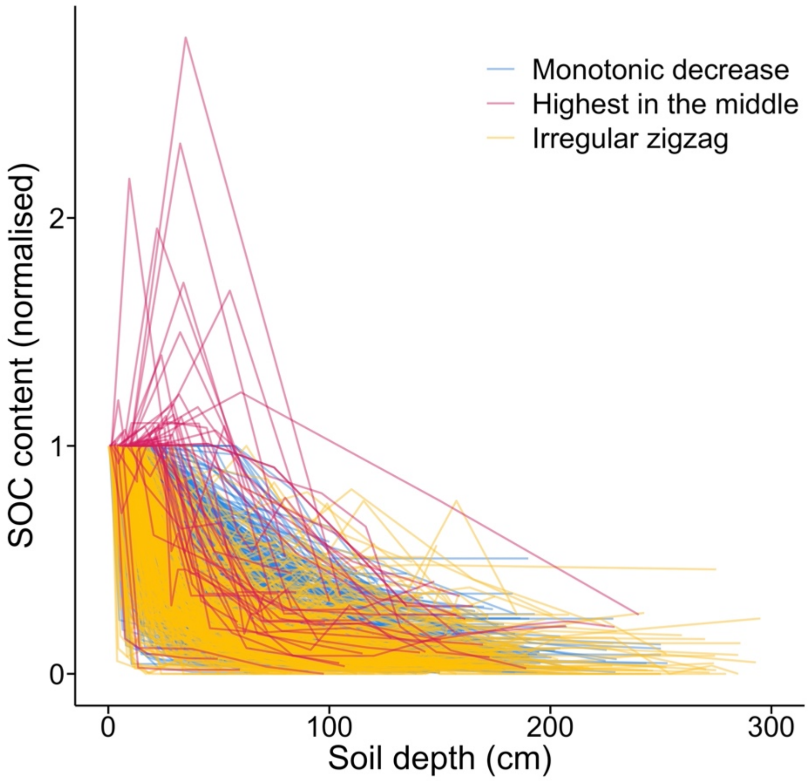

Supplementary Fig. 5 | Different vertical shapes of SOC profiles used in this study. Shown in the figure are 1,000 profiles randomly selected from the 57,276 profiles. SOC values are normalised by the value at the first soil layer of each profile.

Supplementary References

1 Luo, Y. *et al.* Matrix approach to land carbon cycle modeling. *Journal of Advances in Modeling Earth Systems*, e2022MS003008, doi:https://doi.org/10.1029/2022MS003008 (2022).

2 Li, J., Chen, L., Zhang, J. & Zhuo, Y. Relationship Between Soil Organic Carbon and Microbial Community in Lime Concrete Black Soil. Soils, 2019, . *Soils* **51**, 488-494 (2019).

3 Wang, C. *et al.* Large-scale importance of microbial carbon use efficiency and necromass to soil organic carbon. *Global Change Biology* (2021).

4 Liu, Z. *et al.* Greater microbial carbon use efficiency and carbon sequestration in soils: Amendment of biochar versus crop straws. *GCB Bioenergy* **12**, 1092-1103 (2020).

5 Spohn, M., Klaus, K., Wanek, W. & Richter, A. Microbial carbon use efficiency and biomass turnover times depending on soil depth–Implications for carbon cycling. *Soil Biology and Biochemistry* **96**, 74-81 (2016).

6 Spohn, M. *et al.* Soil microbial carbon use efficiency and biomass turnover in a long-term fertilization experiment in a temperate grassland. *Soil Biology and Biochemistry* **97**, 168-175 (2016).

7 Poeplau, C. *et al.* Increased microbial anabolism contributes to soil carbon sequestration by mineral fertilization in temperate grasslands. *Soil Biology and Biochemistry* **130**, 167-176 (2019).

8 Liu, W., Qiao, C., Yang, S., Bai, W. & Liu, L. Microbial carbon use efficiency and priming effect regulate soil carbon storage under nitrogen deposition by slowing soil organic matter decomposition. *Geoderma* **332**, 37-44 (2018).

9 Schroeder, J. *et al.* Carbon use efficiency and microbial functional diversity in a temperate Luvisol and a tropical Nitisol after millet litter and N addition. *Biology and Fertility of Soils* **56**, 1139-1150 (2020).

10 Zheng, Q. *et al.* Growth explains microbial carbon use efficiency across soils differing in land use and geology. *Soil Biology and Biochemistry* **128**, 45-55 (2019).

11 Walker, T. W. *et al.* Microbial temperature sensitivity and biomass change explain soil carbon loss with warming. *Nature climate change* **8**, 885-889 (2018).

12 Chen, X. *et al.* Microbial carbon use efficiency, biomass turnover, and necromass accumulation in paddy soil depending on fertilization. *Agriculture, Ecosystems & Environment* **292**, 106816 (2020).

13 Widdig, M. *et al.* Microbial carbon use efficiency in grassland soils subjected to nitrogen and phosphorus additions. *Soil Biology and Biochemistry* **146**, 107815 (2020).

14 Zhran, M. *et al.* Assessment of depth‐dependent microbial carbon‐use efficiency in long‐term fertilized paddy soil using an 18O–H2O approach. *Land Degradation & Development* **32**, 199-207 (2021).

15 Oliver, E. E., Houlton, B. Z. & Lipson, D. A. Controls on soil microbial carbon use efficiency over long-term ecosystem development. *Biogeochemistry* **152**, 309-325 (2021).

16 Li, J. *et al.* Stoichiometric imbalance and microbial community regulate microbial elements use efficiencies under nitrogen addition. *Soil Biology and Biochemistry* **156**, 108207 (2021).

17 Schroeder, J., Kammann, L., Helfrich, M., Tebbe, C. C. & Poeplau, C. Impact of common sample pre-treatments on key soil microbial properties. *Soil Biology and Biochemistry*, 108321 (2021).

18 Hengl, T. *et al.* SoilGrids250m: Global gridded soil information based on machine learning. *PLoS one* **12**, e0169748 (2017).

19 Beck, H. E. *et al.* Present and future Köppen-Geiger climate classification maps at 1-km resolution. *Scientific data* **5**, 180214 (2018).

20 Fick, S. E. & Hijmans, R. J. WorldClim 2: new 1‐km spatial resolution climate surfaces for global land areas. *International Journal of Climatology* **37**, 4302-4315 (2017).
